# Mechanical Thrombectomy for Basilar Artery Occlusion Stroke: A Case Report

**DOI:** 10.31729/jnma.5174

**Published:** 2020-08-31

**Authors:** Subash Phuyal, Kapil Dawadi, Raju Paudel, Ritesh Lamsal, Pooja Agrawal

**Affiliations:** 1 Department of Neuroimaging and Interventional Neuroradiology, Grande International Hospital, Kathmandu, Nepal; 2 Department of Radiology, Grande International Hospital, Kathmandu, Nepal; 3 Department of Neurology, Grande International Hospital, Kathmandu, Nepal; 4 Department of Anaesthesiology, Tribhuvan University Teaching Hospital, Kathmandu, Nepal; 5 Department of Radiology, Norvic International Hospital, Kathmandu, Nepal

**Keywords:** *basilar artery*, *case report*, *posterior circulation*, *stroke*, *thrombectomy*

## Abstract

Posterior circulation strokes are potentially devastating events that carry a significant risk of morbidity and mortality. Acute basilar artery occlusion stroke is a rare posterior circulation stroke that needs emergent management. We report the case of a 67-year-old woman who developed an acute basilar artery occlusion. We achieved complete recanalization of the occluded basilar artery and its branches with endovascular mechanical thrombectomy. It is possible to achieve excellent results with mechanical thrombectomy in acute basilar artery occlusion if timely diagnosis and reperfusion can be done. We are not aware of any previous publication from Nepal describing this technique in acute basilar artery occlusion.

## INTRODUCTION

Acute basilar artery occlusion (BAO) is a devastating subtype of posterior circulation stroke. It carries a risk of significant morbidity and mortality.^1^ Intravenous thrombolysis is the conventional standard-of-care in eligible patients with acute BAO and other strokes of the posterior circulation; however, thrombolysis alone may not yield satisfactory reperfusion in many patients.^1^ Newer evidences suggest that mechanical thrombectomy (MT) improves recanalization and clinical outcome in posterior circulation strokes. Here, we report a patient with an acute BAO who was successfully treated with MT. We are not aware of any previous publication from Nepal describing this technique in acute BAO.

## CASE REPORT

**Figure 1.**
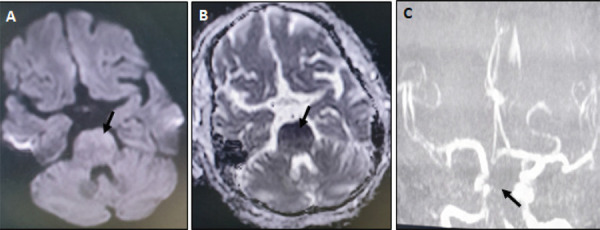
(A, B) Axial diffusion-weighted image and apparent diffusion coefficient map show diffusion restriction on the anterior aspect of the pons (black arrows) (C) TOF MR-angiography shows nonvisualization of the basilar artery (black arrow) and its branches.

**Figure 2.**
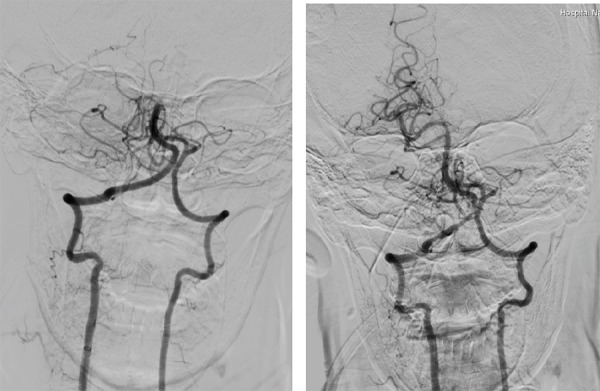
(A) A 2-D left vertebral artery run shows complete cut-off of the proximal basilar artery. (B) Final angiogram shows complete recanalization of the occluded basilar artery and its branches.

A 67-year-old woman presented with weakness of the right side of the body and left-sided facial deviation for five hours. She had hypertension for five years and atrial fibrillation for 20 days, for which she was taking regular medications. She had no remarkable past medical history. On magnetic resonance imaging and magnetic resonance angiography, we found an acute infarct in the anterior part of the pons with complete occlusion of the entire basilar artery (BA) from its origin (Figure 1). After written informed consent, contact aspiration thrombectomy was planned. Right femoral access was obtained and a 6-F guiding catheter (Neuron Max, Penumbra, USA) was placed into the left proximal vertebral artery. The diagnostic angiogram showed a complete cut-off of the proximal BA with non-visualization of the distal branches (Figure 2A). A large-bore aspiration catheter (ACE 68 reperfusion catheter, Penumbra, USA)was advanced through the guiding catheter over a 0.035 inch 150 cm guidewire (Radifocus Guidewire M, Terumo Medical Corporation, Japan). A microcatheter/microguidewire assembly (Headway 27/Traxcess 14, MicroVention, USA) was advanced through the aspiration catheter into the occluded artery. The aspiration catheter was advanced over the microcatheter/microguidewire assembly till the level of the clot and thrombo-aspiration was performed. The occluded BA was recanalized after a single pass. The check-angiogram showed complete recanalization of the BA and its distal branches (Figure 2B).

## DISCUSSION

Posterior circulation strokes constitute only one-fifth of all acute ischemic stroke (AIS) events; however, they cause substantial morbidity and mortality.^1^ In contrast to posterior circulation strokes, MT has been established through several international randomized trials in anterior circulation strokes. As there is a dearth of high-quality studies in posterior circulation stroke, standard therapeutic protocols or devices have not been uniformly established or adopted. The various treatment modalities that have been tried or are available for posterior circulation stroke include intravenous thrombolysis, intra-arterial thrombolysis, MT, and stenting or angioplasty if vascular stenosis is present. In recent times, endovascular techniques are gradually being established as safe and effective, resulting in good clinical outcomes in posterior circulation strokes.^2^

BA strokes are rare posterior circulation events that account for only around 1% of all reported strokes.^3^ A vexing problem with BAO is that clinical presentations are highly variable, often delaying rapid diagnosis and subsequent revascularization therapy.^4^ Among patients treated conventionally with antiplatelets or anticoagulants, there is a high rate of poor outcomes in patients with BAO despite recent advances in stroke management.^1^ The Basilar Artery International Cooperation Study (BASICS) did not find the superiority of any one of the three treatment strategies for BAO -antithrombotics (antiplatelets or systemic anticoagulants), primary intravenous thrombolysis, and intra-arterial therapy (intra-arterial thrombolysis, mechanical thrombectomy, stenting, or a combination of these).^1^ However, the findings of the BASICS study should be interpreted with caution as it was an observational study, and high-quality endovascular devices were not available to perform MT. The ENDOSTROKE study^5^ found that MT using a stent-retriever is associated with a high recanalization rate (79%), but recanalization alone does not accurately predict clinical outcome. Age, hypertension, stroke severity, collateral status, and the type of imaging modality used were independent predictors of clinical outcomes.^5^ In a recently published study, Gory et al^6^ compared the two common MT approaches for revascularization of BAO using either direct-aspiration first-pass technique (ADAPT) or stent-retriever devices. They concluded that the ADAPT technique achieves a significantly better complete reperfusion rate (87%) with a shorter duration of the endovascular procedure, and a lower rate of periprocedural complications.^6^ In a smaller study, an even higher complete reperfusion rate (89%) was achieved in patients with BAO with MT techniques.^7^ Even though the ‘gold-standard’ method of revascularization has not been definitively established, evidences strongly favor MT as the most effective treatment of acute BAO.^8^

Acute BAO is a potentially fatal diagnosis, and timely reperfusion is paramount for good clinical outcomes. As the territory of the posterior circulation is very small compared with anterior circulation, even a small infarct can lead to life-threatening complications. Furthermore, diagnostic tools optimized to detect the penumbra zone of the posterior circulation territories are not readily available. Perfusion modalities do not accurately reflect the posterior circulation perfusion and diffusion states. Despite these challenges, we could achieve a complete recanalization of the BA and its distal branches using thrombo-aspiration.

Before the advent of modern endovascular techniques, over 80% of patients with acute BAO receiving conventional treatment reportedly suffered debilitating morbidity.^9^ These numbers have greatly improved in recent times. We do not have proper data regarding the clinical profile of patients with acute BAO from Nepal; however, with the recent development of neurointervention facilities in the country, it is possible to achieve excellent outcomes, if early diagnosis and timely revascularization can be done.

## Consent:

**JNMA Case Report Consent Form** was signed by the patient and the original article is attached with the patient's chart.

## Conflict of Interest

**None.**
